# Using Innovative Machine Learning Methods to Screen and Identify Predictors of Congenital Heart Diseases

**DOI:** 10.3389/fcvm.2021.797002

**Published:** 2022-01-07

**Authors:** Yanji Qu, Xinlei Deng, Shao Lin, Fengzhen Han, Howard H. Chang, Yanqiu Ou, Zhiqiang Nie, Jinzhuang Mai, Ximeng Wang, Xiangmin Gao, Yong Wu, Jimei Chen, Jian Zhuang, Ian Ryan, Xiaoqing Liu

**Affiliations:** ^1^Guangdong Cardiovascular Institute, Guangdong Provincial People's Hospital, Guangdong Academy of Medical Sciences, Guangzhou, China; ^2^Department of Environmental Health Sciences, University at Albany, State University of New York, New York, NY, United States; ^3^Department of Biostatistics and Bioinformatics, Rollins School of Public Health, Emory University, Atlanta, GA, United States

**Keywords:** congenital heart disease, machine learning, prediction, laboratory tests, clinical indicators

## Abstract

**Objective:** Congenital heart diseases (CHDs) are associated with an extremely heavy global disease burden as the most common category of birth defects. Genetic and environmental factors have been identified as risk factors of CHDs previously. However, high volume clinical indicators have never been considered when predicting CHDs. This study aimed to predict the occurrence of CHDs by considering thousands of variables from self-reported questionnaires and routinely collected clinical laboratory data using machine learning algorithms.

**Methods:** We conducted a birth cohort study at one of the largest cardiac centers in China from 2011 to 2017. All fetuses were screened for CHDs using ultrasound and cases were confirmed by at least two pediatric cardiologists using echocardiogram. A total of 1,127 potential predictors were included to predict CHDs. We used the Explainable Boosting Machine (EBM) for prediction and evaluated the model performance using area under the Receive Operating Characteristics (ROC) curves (AUC). The top predictors were selected according to their contributions and predictive values. Thresholds were calculated for the most significant predictors.

**Results:** Overall, 5,390 mother-child pairs were recruited. Our prediction model achieved an AUC of 76% (69-83%) from out-of-sample predictions. Among the top 35 predictors of CHDs we identified, 34 were from clinical laboratory tests and only one was from the questionnaire (abortion history). Total accuracy, sensitivity, and specificity were 0.65, 0.74, and 0.65, respectively. Maternal serum uric acid (UA), glucose, and coagulation levels were the most consistent and significant predictors of CHDs. According to the thresholds of the predictors identified in our study, which did not reach the current clinical diagnosis criteria, elevated UA (>4.38 mg/dl), shortened activated partial thromboplastin time (<33.33 s), and elevated glucose levels were the most important predictors and were associated with ranges of 1.17-1.54 relative risks of CHDs. We have developed an online predictive tool for CHDs based on our findings that may help screening and prevention of CHDs.

**Conclusions:** Maternal UA, glucose, and coagulation levels were the most consistent and significant predictors of CHDs. Thresholds below the current clinical definition of “abnormal” for these predictors could be used to help develop CHD screening and prevention strategies.

## Introduction

Congenital heart diseases (CHDs) rank first among birth defects worldwide and are emerging as a global problem in child health (1). According to the 2017 Global Burden of Disease Study, more than 11 million individuals live with CHDs globally, and CHDs have caused approximately 89 thousand years lived with disability (1). This implies a tremendous economic burden for both the affected families and the whole society. However, to date, no public policies or interventions are specifically directed at reducing the impact of CHDs. Over the past decades, there have been breakthroughs in understanding the inherited causes of CHDs, including identifying specific genetic abnormalities for selected CHD phenotypes (2, 3). There is also a growing body of epidemiological research identifying non-inherited risk factors for CHDs, including maternal socioeconomic status (SES), illness, therapeutic and non-therapeutic drug exposures, environmental exposures, and paternal exposures (4). These risk factors that previous studies identified explained the causes of approximately 20-30% of CHD cases (5). Most CHDs, therefore, remain unexplained and are presumed to be multi-factorial.

There are still several knowledge gaps in this area of research, which implies new opportunities to predict and prevent CHDs. First, most of the known predictors were derived from case-control studies. Self-reported questionnaires obtained information on parental exposures after the child was born, which may suffer from recall or reporting biases. Second, although previous epidemiological studies indicated dozens of risk factors for CHDs (4), the role of maternal routine laboratory test results during the critical window for fetal heart development has seldom been studied. These indicators are routinely collected in clinical practice and consume time and money to be maintained yet have never been used in CHD research before. Third, combining objective clinical indicators with self-reported variables to develop a CHD predictive model could provide unique opportunities to develop efficient screening and prevention tools in clinical settings that deal with prenatal care. Finally, high collinearities exist among some of these high-volume variables. Under such conditions, traditional statistical methods are not adequate to predict CHDs with a non-converge model. Machine learning (ML) provides a potential way to solve this issue.

Compared to traditional models, ML algorithms usually have significantly higher predictive performance and are robust in the presence of collinearity problems and outliers. An increasing number of studies in the field of heart disease now use ML algorithms (6). ML approaches have also been utilized to identify and predict CHDs. Most of these studies tried to identify CHDs based on heart sounds, images, ECG, and genetic makeup (7, 8). Three previous studies predicted the occurrence of CHDs based on a limited number of self-reported factors from questionnaires (9–11). One was a hospital-based case-control study involving 15 significant self-reported variables from interviews conducted with 119 CHD cases and 239 controls from central China (9). The other two studies used the same dataset from a retrospective cross-sectional study from northwest China that included nine composite variables collected via questionnaire (*n* = 78 CHD cases) (10, 11). Unfortunately, none of these studies considered clinical laboratory indicators to predict CHDs.

To fill the knowledge gaps described above, we conducted a birth cohort study to identify the most important predictors of CHDs and their predictive characteristics, including area under the Receiver Operating Characteristic (ROC) curves (AUC), accuracy, sensitivity, specificity, and thresholds for a total of 1,127 variables using ML algorithms, specifically the Explainable Boosting Machine (EBM). These variables were gathered through self-reported questionnaires and routine clinical laboratory data collected during early pregnancy.

## Materials and Methods

### Study Design and Participants

This birth cohort study was conducted at a cardiac referral center in Guangdong province, south China from 2011 to 2017. All pregnant women accepting prenatal care in the Department of Obstetrics and Gynecology, with informed consent, were included at their first interview to create the profile of pregnancy and delivery around the 16th gestational week. Our inclusion and exclusion criteria were as follows. (1) Births with chromosomal anomalies, gene mutations, or non-cardiac defects were excluded as recommended by EUROCAT to focus on isolated CHDs (12). (2) We also excluded twins and multiple births because they might possess different etiology from singletons (13). Finally, (3) if a mother was enrolled in the cohort multiple times during different pregnancies, only the first record was included because ML methods usually cannot control for clustering.

### Health Outcomes and Data Sources

CHDs were the primary outcome of interest in our study. Fetuses were screened for congenital abnormalities by basic ultrasound at 11-13 and 15-20 gestational weeks. Fetuses suspected of having a CHD underwent an echocardiogram. Every newborn was clinically evaluated for congenital abnormalities before discharge (usually within 72 h of birth). Each CHD case was confirmed by at least two senior pediatric cardiologists using a post-natal echocardiogram. A third senior pediatric cardiologist resolved any disagreements. Results of computed tomography, cardiac catheterization, surgery, or autopsy were referenced to assist the diagnosis. CHD diagnoses were coded with detailed phenotypes according to the International Classification of Diseases version 10 (ICD-10) with codes Q20-Q28.

### Predictors and Data Collection

We obtained a total of 1,127 variables from self-reported questionnaires and routine clinical laboratory test results. The timing of when different variables were collected for each participant is illustrated in Supplementary Figure 1. We collected self-reported variables through face-to-face interviews at the time of enrollment conducted by two obstetric nurses using a standard, structured questionnaire. These variables included parental SES, disease status, medication use, behavior, reproductive history, and environmental exposures during periconceptional periods (from 6 months before pregnancy to the time of interview). Meanwhile, we extracted the first-time record of routine laboratory test results from medical charts. The Department of Laboratory Medicine performed all the clinical tests in our hospital according to the current standardized protocol for systematically used testing in hospitals. The enrolled women were followed for their entire pregnancy. At the time of hospitalization for delivery or termination, the second face-to-face interview was conducted to evaluate maternal exposures between the first interview and the end of pregnancy. In addition, outcomes and basic information regarding the newborns were recorded.

### Statistical Analysis

We first excluded variables with missing rates higher than 50% and some categorical variables, almost all with only one category. Then, we used the chi-square test to evaluate the difference in CHD incidence among groups of participants by characteristics. In addition, the *t*-test was used to detect the difference in distributions of maternal continuous laboratory test results between CHD cases and non-CHD mother-infant pairs.

Second, we used a stratified approach to randomly split the dataset into two parts: a training set (70%) and a testing set (30%). The training set was used in the model training process, and the testing set was used for the final validation. To deal with the imbalance problem in the training set due to the low incidence of CHDs, we applied the AllKNN method from SMOTE approaches to balance the data (14). The AllKNN approach first builds a KNN model and computes several nearest neighbor centers. Then, it under samples the majority class (non-CHD in this study) by removing samples with different classes from their nearest neighbor centers. This approach dramatically reduces the unbalanced ratio represented in the sample. We then incorporated a continuous random term and a categorical random term into the EBM to identify the importance score as the thresholds to select significant variables. Variables with importance scores greater than their corresponding random terms were included in the final EBM to predict CHDs in the training set. Afterward, we trained the final model by only including the selected significant variables using cross-validation to select the best hyperparameters. Finally, the performance of this final model was evaluated in the testing set.

In this study, we used EBM to predict the occurrence of CHDs. EBM is derived from the generalized additive model (GAM) and uses techniques from Random Forest and Boosted Tree models. The basic form of EBM is:

$$\begin{array}{l} g\left(E\left[y\right]\right)=\beta_{0}+\sum f_{j}\left(x_{j}\right) \end{array}$$

Where *g* is the link function for classification and *f* is the feature function. The major differences between EBM and traditional GAMs include: (1) Each feature function *fj* in EBM is determined using modern ML techniques, such as bagging and gradient boosting, with round-robin cycles; (2) EBM can automatically detect and include pairwise interaction terms and improves accuracy; (3) EBM plots the *f* feature function to examine the association between each variable and the outcome. Previous studies suggest that EBM performs better on health datasets than other established ML models, including the light gradient boosting model, regularized logistic regression, random forest, and xgboost (15). To verify EBM's performance using our data, we conducted additional analyses using six other ML models, including random forest, gradient boosting, Xgboost, logistic regression, ANN, and Naïve Bayesian (Supplementary Table 1). We also built a Vote Classifier by combining all these seven models (EBM, random forest, gradient boosting, Xgboost, logistic regression, ANN, and Naïve Bayesian). EBM outperformed all other approaches and the combined Voting classifier on our data.

Finally, we evaluated model performance within the testing set using the ROC curves. Commonly used performance indicators including AUC, accuracy, sensitivity and specificity were calculated. We also considered F-score and the Precision-Recall curve when assessing the performance of our model. F-score is a harmonic indicator considering both precision and recall as:

$$\begin{array}{l} F-score=\left(1+\beta^{2}\right)\frac{Precision\cdot Recall}{\beta^{2}\cdot Precision+Recall} \end{array}$$

Where precision and recall are two indexes commonly used to assess the performance of ML models: $Precision=\frac{True\;positive}{True\;positive+False\;positive}$; $Recall=\frac{True\;positive}{True\;positive+False\;negtive}$. If β = 1, we get an F1-score, and at this time, the precision and recall are equally important and equally weighted. When β = 2, we get F2 score and recall has a higher weight and is more important at this point. We compared the predictive performance of the predictors by calculating AUC. We ranked the top 35 predictors of CHDs according to their contributions in the model. An online predictive tool for CHDs was established based on our model to assist in developing prevention and screening strategies. For the top predictors, their associations with CHDs were assessed individually after controlling for the other predictors. For continuous predictors, their linear and non-linear associations with CHDs were simulated by fitting loess models, and the thresholds were defined by optimizing the Youden index. We categorized the study participants into low-risk and high-risk groups according to the thresholds. We then calculated the risk ratios (RRs) of CHDs in the high-risk groups compared to low-risk groups using normal distribution approximation (16, 17). Predictive values, including AUC, accuracy, sensitivity, and specificity were also calculated for each of the most significant predictors. A two-side *P*-value < 0.05 was considered statistically significant. We accomplished all analyses using Python 3.7 and R 3.6.1 (R Core Team, 2019).

## Results

### Basic Characteristics of Study Participants

Overall, we included 5,390 mother-child pairs, and among them, 157 (2.9%) babies with CHDs were identified (Supplementary Figure 2). Detailed phenotypes of CHDs we diagnosed were presented in Supplementary Table 2. Our study participants' birth outcomes and maternal characteristics are presented in Supplementary Tables 3, 4, respectively. Maternal clinical laboratory indicators were tested at gestational week 16 (median) (range: 7-28). In total, we included 1,127 variables in the ML models (Supplementary Table 5). Among them, 379 were obtained from the self-reported questionnaire, 699 from routine clinical laboratory test data, and 49 were extracted from infants' medical charts.

### Prediction of CHDs Occurrence

**Figure 1:**
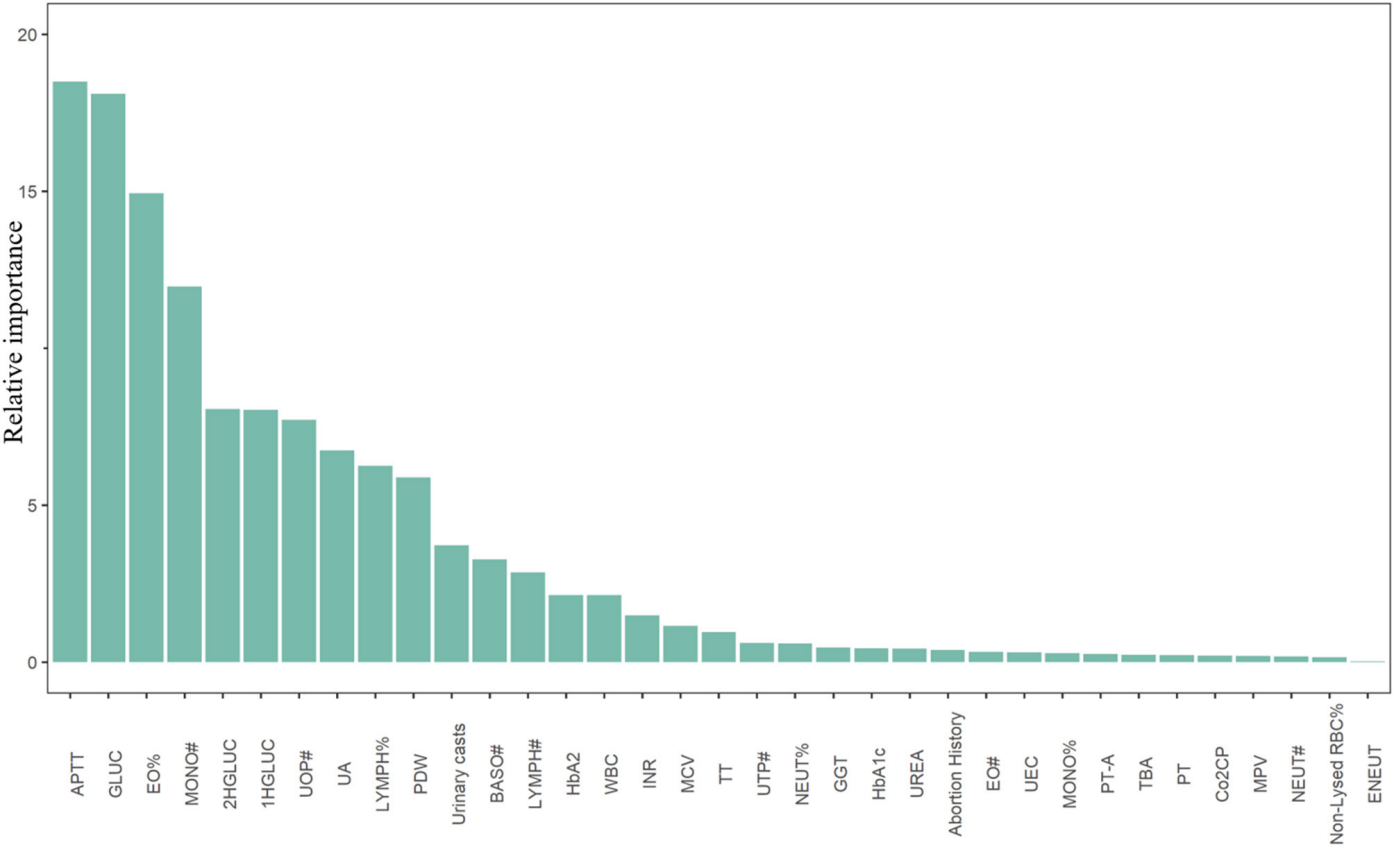
Top 35 predictors of congenital heart diseases derived from the Explainable Boosting Machine. APTT, activated partial thromboplastin time; GLUC, fasting plasma blood glucose; EO%, eosinocyte ratio; MONO#, monocyte counts; 2HGLUC, 2-h plasma blood glucose; 1HGLUC, 1-h plasma blood glucose; UOP#, uric other particles counts; UA, serum uric acid; LYMPH%, lymphocyte ratio; PDW, platelet distribution width; BASO#, basophilic granulocyte count; LYMPH#, lymphocyte count; HbA2, hemoglobin A2; WBC, white blood cells; INR, international normalized ratio; MCV, mean corpuscular volume; TT, thrombin time; UTP#, uric total particles counts; NEUT%, neutrocyte ratio; GGT, γ-glutamyl transpeptidase; HbA1c, hemoglobin A1c; UREA, serum urea; EO#, eosinocyte count; UEC, urine epithelial cells; MONO%, monocyte ratio; PT-A, prothrombin time activity percentage; TBA, total bile acid; PT, prothrombin time; Co2CP, carbon dioxide combining power; MPV, mean platelet volume; NEUT#, neutrocyte count; Non-Lysed RBC%, percentage of non-lysed red blood cell; ENEUT, elevated neutrocyte.

Using the data derived from above, an EBM was trained to predict CHD occurrence. The model achieved an AUC of 76% (69-83%) in the testing sample (Supplementary Figure 3). The F1, F2 score, and Precision-Recall curve of our model were shown in Supplementary Figures 4-6. By using 0.05 as the cut-off value, our model could obtain a total accuracy, sensitivity, and specificity of 0.94, 0.26 and 0.96, respectively. The F1 and F2 score was about 0.2 and 0.25, respectively. However, for CHDs with a high disease burden, sensitivity is the most important indicator when selecting the cutoff value of our model. Thus, we defined 0.03 with the relative high sensitivity as the cutoff value and got a total accuracy, sensitivity, and specificity of 0.65, 0.74, and 0.65, respectively (Table 1). Meanwhile, we obtained a F1 score of 0.1, F2 score of 0.22, and the area under the Precision-Recall curve of 0.15 (Supplementary Figures 4-6). The sensitivity (0.72) and specificity (0.61) from the combined Voting classifier did not improve those of our EBM. The top 35 predictors of CHDs identified in our study are shown in Figure 1. Detailed information regarding predictor scores and definitions are presented in Supplementary Table 6. All the top 35 predictors of CHDs were obtained from laboratory tests except for self-reported abortion history, which ranked 24th among predictors. Among the top predictors of CHDs, maternal coagulation function indicators [APTT, international normalized ratio (INR), thrombin time (TT), and prothrombin activity (PT-A)], glucose levels [fasting plasma glucose (FPG), 1-h plasma glucose (PG), and 2-h PG] and maternal serum UA levels were the most consistently important.

**Table 1:** Predictive values of maternal uric acid, glucose, and coagulation levels during early pregnancy for congenital heart diseases.

**Variables**	Thresholds^*^	**Sensitivity**	**Specificity**	**Accuracy**	**AUC**
EBM model	0.03	0.74	0.65	0.65	0.76 (0.69, 0.83)
Maternal serum UA	261.13 umol/L	0.52	0.71	0.70	0.63 (0.58,0.67)
Maternal fasting plasma glucose	4.35 mmol/L	0.85	0.32	0.34	0.55 (0.51,0.59)
Maternal 1-h plasma glucose	6.14 mmol/L	0.78	0.32	0.33	0.53 (0.49,0.56)
Maternal 2-h plasma glucose	7.01 mmol/L	0.86	0.28	0.30	0.53 (0.50,0.57)
Maternal APTT	33.33 s	0.64	0.52	0.53	0.57 (0.53,0.62)
Maternal INR	1.01	0.95	0.10	0.12	0.51 (0.47,0.56)
Maternal TT	14.64 s	0.32	0.75	0.74	0.52 (0.48,0.57)
Maternal PT-A	98.67	0.94	0.11	0.14	0.51 (0.47,0.56)

*AUC, area under the curve*.

* *Thresholds with optimal Youden index to predict CHDs*.

### Associations Between Clinical Indicators and CHDs

**Figure 2:**
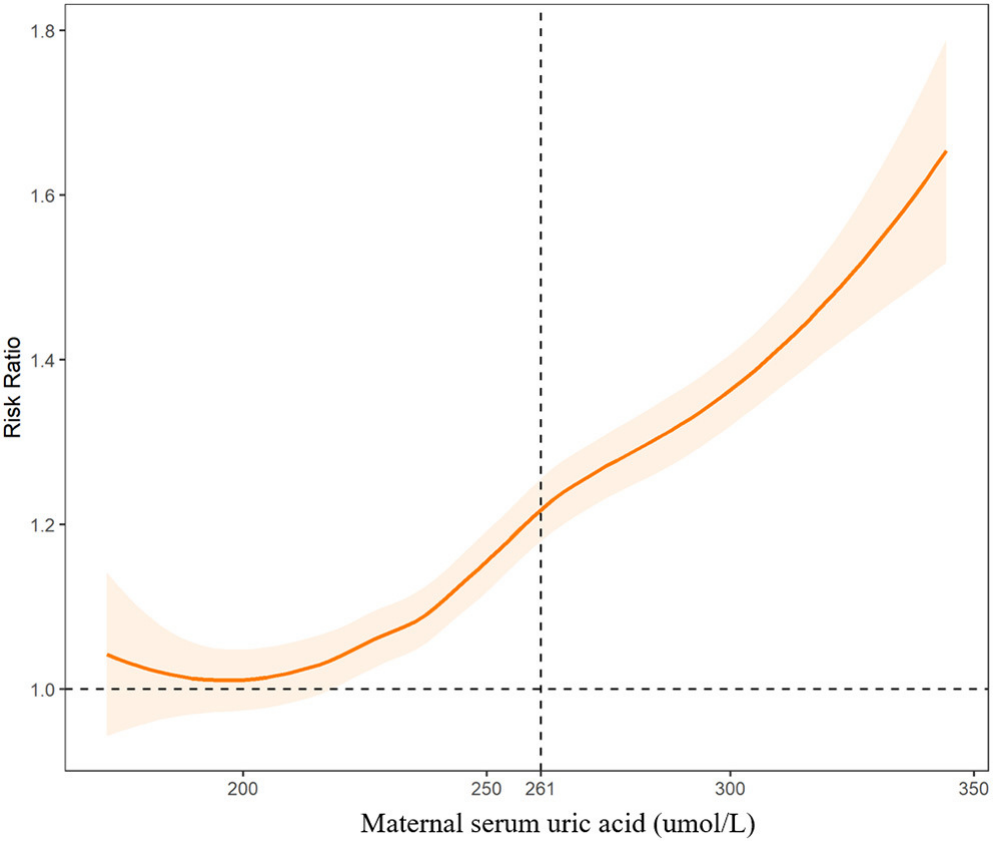
Associations between maternal serum uric acid levels and risk of congenital heart diseases in offspring. The dashed line indicates the threshold with the optimal Youden index to predict CHDs.

**Figure 3:**
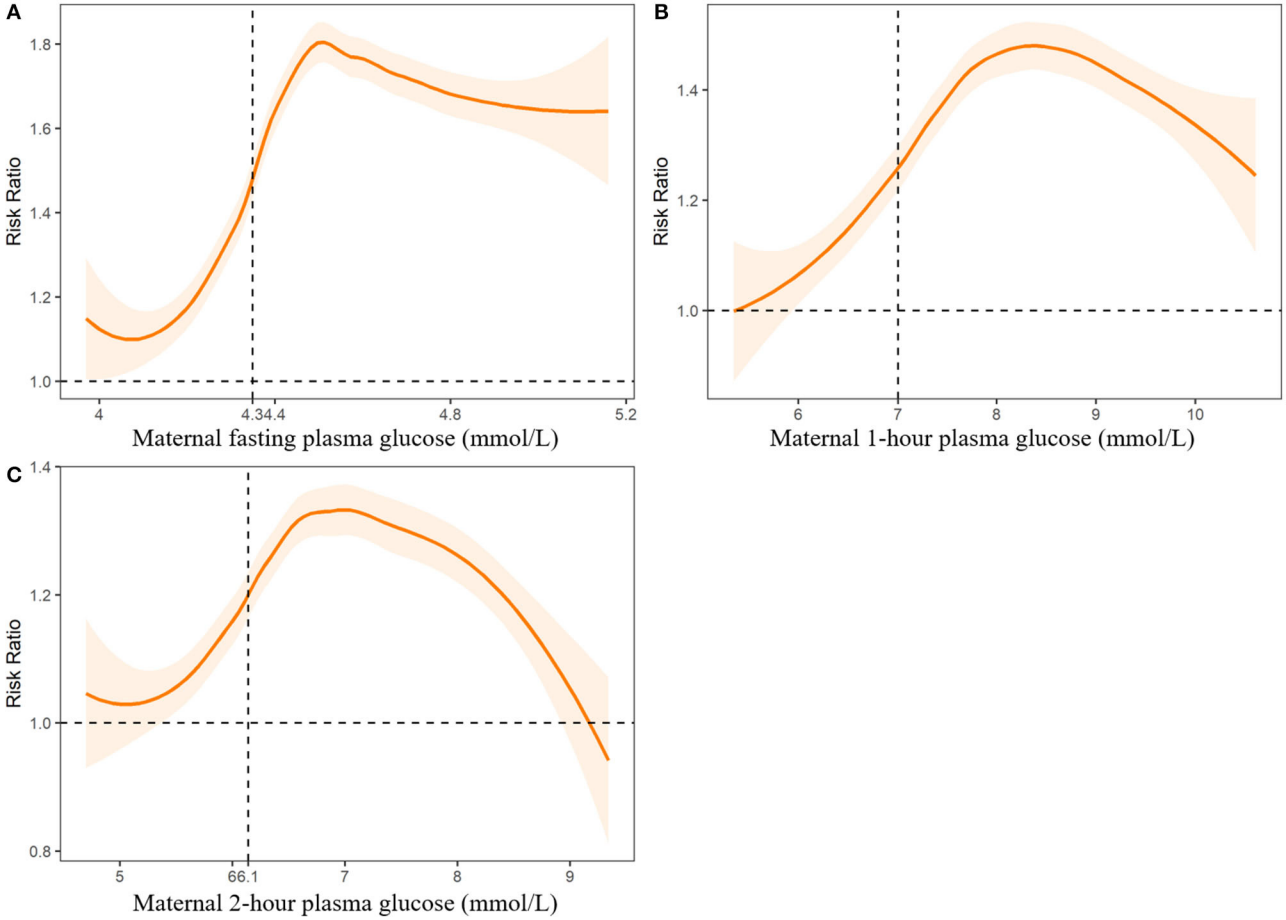
Associations between maternal plasma fasting plasma glucose **(A)**, 1-h plasma glucose **(B)** and 2-h plasma glucose levels **(C)** and risk of congenital heart diseases in offspring. Dashed lines indicate the thresholds with optimal Youden index to predict CHDs.

**Figure 4:**
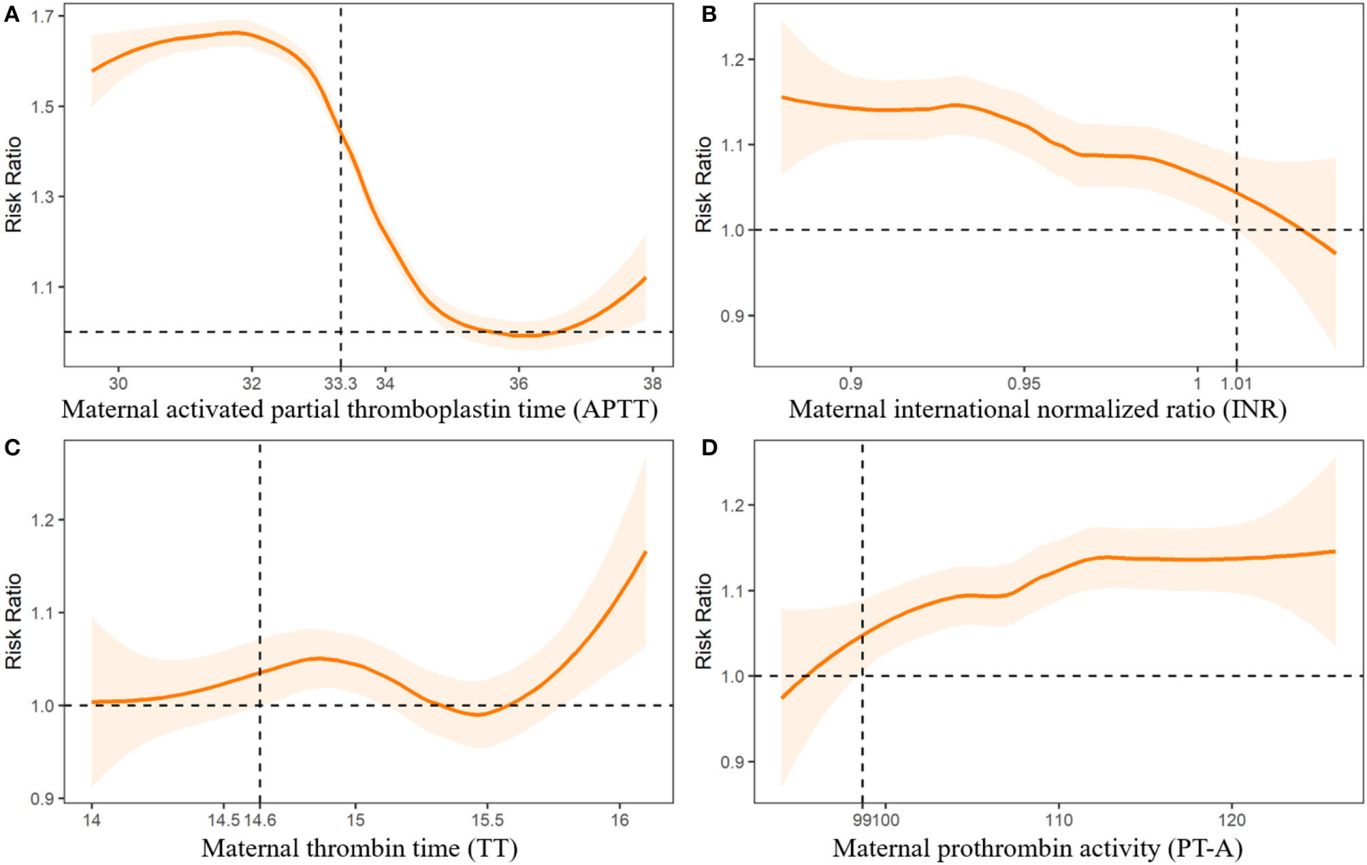
Associations between maternal coagulation indexes and risk of congenital heart diseases in offspring. Dashed lines indicate the thresholds with optimal Youden index to predict CHDs.

We further fitted the relationships between maternal UA, glucose, and coagulation levels with CHDs by controlling for the other predictors of CHDs. The results are presented in Figures 2–4. As shown in Figure 2, the risk of CHDs increased almost monotonically with elevated UA levels in early pregnancy. There were consistent inverse U-shaped relationships between FPG, 1-h PG, and 2-h PG levels and CHD risks (Figure 3). There was an unexpected decreased risk of CHDs (RR <1) when maternal 2-h plasma glucose PG ≥ 9 mmol/L. This might be due to lower precision or inadequate study power as a result of the limited number of mothers with 2-h PG levels higher than 9 mmol/L (*N* = 372, 6.9%), especially in CHD cases (*N* = 4). For the relationships between maternal coagulation indexes and CHDs, we observed that the risk of CHDs increased with shortened APTT, lower INR (Figures 4A,B), and elevated PT-A (Figure 4D). There was no apparent relationship between TT and CHD risk (Figure 4C).

### Thresholds of Clinical Indicators to Predict CHDs

In Figures 2-4, we marked the thresholds of maternal UA, glucose, and coagulation levels with optimal Youden indexes to predict CHDs using dashed lines. The threshold of UA to predict CHDs was 261.13 umol/L (4.38 mg/dl). For glucose levels, the threshold of FPG, 1-h PG, and 2-h PG levels to predict CHDs was 4.35, 6.14, and 7.01 mmol/L, respectively. The thresholds of APTT, INR, TT, and PT-A to predict CHDs were 33.33, 1.01, 14.64, and 98.67 s, respectively.

Detailed thresholds and predictive values for UA, glucose, and coagulation levels are shown in Table 1. UA, followed by APTT, was the most valuable predictor with the highest AUC and acceptable accuracy, sensitivity, and specificity. Predictive values of the three indicators of glucose (FPG, 1-h PG, and 2-h PG) for CHDs were consistent with high sensitivity and acceptable AUC, but low specificity. Although INR and PT-A obtained the highest sensitivity (0.94-0.95), they also brought the highest false-positive risk with the lowest specificity (0.10-0.11).

### Risks of CHDs Associated With Abnormal Clinical Indicators

We defined abnormal UA, glucose, and coagulation levels according to the thresholds above and evaluated the associated RRs of CHDs (Table 2). We found that FPG > 4.35 mmol/L was associated with the highest risk of CHDs (aRR = 1.54, 95%CI: 1.49-1.59), followed by APTT < 33.33 s (aRR = 1.42, 95%CI: 1.39-1.46), 2-h PG > 7.01 mmol/L (aRR = 1.29, 95%CI: 1.25-1.33), UA > 261.13 umol/L (aRR = 1.27, 95%CI: 1.24-1.31), INR < 1.01 (aRR = 1.19, 95%CI: 1.17-1.22), 1-h PG > 6.14 mmol/L (aRR = 1.18, 95%CI: 1.15-1.20), and PT-A > 98.67 (aRR = 1.17, 95%CI: 1.15-1.19), respectively.

**Table 2:** Risk ratios of congenital heart diseases with exposure to abnormal uric acid, glucose and coagulation levels.

**Variables**	Abnormal condition^*^	**Current clinical reference criteria for normal**	**aRRs (95%CI)**
Maternal serum UA	>261.13 umol/L (4.38 mg/dl)	<360 umol/L (6 mg/dl)	1.27 (1.24,1.31)
Maternal fasting blood glucose	>4.35 mmol/L	<5.1 mmol/L	1.54 (1.49,1.59)
Maternal 1-hour glucose	>6.14 mmol/L	<10.0 mmol/L	1.18 (1.15,1.20)
Maternal 2-hour glucose	>7.01 mmol/L	<8.5 mmol/L	1.29 (1.25,1.33)
Maternal APTT	<33.33 s	20-40 s	1.42 (1.39,1.46)
Maternal INR	<1.01	0.8-1.5	1.19 (1.17,1.22)
Maternal TT	>14.64 s	11-14 s	1.02 (1.00,1.04)
Maternal PT-A	>98.67	75-100%	1.17 (1.15,1.19)

*Adjusted for all the variables in the model*.

*UA, uric acid; APTT, activated partial thromboplastin time; INR, international normalized ratio; TT, thrombin time; PT-A, prothrombin time activity percentage*.

* *According to the thresholds with optimal Youden index to predict CHDs in the current study*.

## Discussion

### Predictors of CHDs

Our results indicated that maternal routine laboratory test results during early pregnancy might be more important for predicting CHDs than self-reported variables from questionnaires. We included more than 1,000 variables from both questionnaires and laboratory test results. However, only one predictor obtained from the questionnaire (abortion history) was selected into the top 35 significant predictors of CHDs. For laboratory test results, we found that maternal UA, glucose levels, coagulation functions, infectious indicators, and other hematuria test results contributed the most. In the current study, we focused on the predicted value of UA, glucose, and coagulation levels for CHDs because the associations between each indicator in these three categories and CHDs were consistently significant. Infection is another composite index that contributes significantly to CHDs. However, it is difficult to define the composition of infection without detailed bacterial or viral isolation results. Thus, we will systematically present the results of infections separately in the future.

None of the previous studies predicting CHD occurrence using ML algorithms considered maternal laboratory test results predictors of CHDs (9, 10). The first hospital-based case-control study involved 119 CHD cases and 239 controls from central China (9). The authors collected 36 variables through face-to-face interviews using questionnaires and used univariate logistic regression analyses to select significant predictors of CHDs. They then developed a standard feed-forward BPNN prediction model for CHDs by involving 15 significant predictors from the univariate logistic regression (gravidity, parity, history of abnormal reproduction, family history of CHD, maternal chronic disease, maternal upper respiratory tract infection, environmental pollution around maternal dwelling place, maternal exposure to occupational hazards, maternal mental stress, paternal chronic disease, and paternal exposure to occupational hazards as risk factors; and high education level, intake of vegetables/fruits, intake of fish/shrimp/meat/egg, and intake of milk/soymilk as protective factors) (9). The other cross-sectional study included 78 CHD cases among 33,831 live births. They compared the accuracy of three classification models [ML algorithms Weighted Support Vector Machine (WSVM), Weighted Random Forest (WRF), and logistic regression (Logit)] in predicting CHDs. Only nine composite variables collected by questionnaires were included in the models (maternal age, annual per capita income, family history, maternal history of illness, nutrition and folic acid deficiency, maternal illness in pregnancy, medication use in pregnancy, environmental risk factors in pregnancy, and unhealthy maternal lifestyle in pregnancy) (10).

Pregnant women are required to undergo several routine laboratory tests during early pregnancy. These laboratory test results are crucial for diagnosing, screening, and monitoring maternal conditions during pregnancy. Combining these objective indicators with self-reported variables to predict CHD occurrence may provide novel opportunities to identify high-risk populations and develop efficient prevention strategies to reduce CHDs. In addition, no additional resources would be required to obtain these test results.

### Maternal Elevated UA Levels and Increased Risk of CHDs

We found that the risk of CHDs increased monotonically with higher UA levels during early pregnancy. The optimal UA threshold to predict CHDs was 261 umol/L (4.38 mg/dl). Compared with mothers with UA ≤ 4.38 mg/dl, those with elevated UA levels (UA > 4.38 mg/dl) suffered a 27% higher risk of having a baby with a CHD.

Consistent with our results, hyperuricemia has been observed as a clinical risk factor for CHDs (18). Ke et al. further studied the role of UA in the process of cardiac differentiation based on their clinical observation that maternal hyperuricemia might be a risk factor for CHDs (18). They confirmed that UA promotes cardiac differentiation of hPSCs and suggested that UA abnormalities might be a risk factor for abnormal fetal heart development in the early stages of embryogenesis. Meanwhile, CHD patients, both cyanotic and non-cyanotic, have higher serum UA levels than patients in the general population (19, 20). In addition, previous epidemiological, experimental, and clinical studies found that hyperuricemia was associated with increased incidence of and mortality related to adult cardiovascular disease (CVD), including hypertension, atherosclerosis, atrial fibrillation, and heart failure (20–22). Hyperuricemia promotes the occurrence and development of CVD by regulating molecular signals, such as inflammatory response, oxidative stress, insulin resistance, endoplasmic reticulum stress, and endothelial dysfunction (21). The associations between elevated UA and CHDs may share similar mechanisms to hyperuricemia and other cardiac diseases.

The threshold of UA to predict CHDs in our study (4.38 mg/dl) was lower than the current clinical criteria defining hyperuricemia in women (6 mg/dl) (22). Another study defined hyperuricemia as female UA levels exceeding 5.7 mg/Dl (23). However, consistent with our study, a recent study confirmed that the threshold of UA levels to increase total mortality (4.7 mg/dl) and cardiovascular mortality (5.6 mg/dl) risk were also significantly lower than the current clinical diagnostic criteria (24). Considering the free transfer of UA *via* the placenta (25), higher UA exposure, even below the hyperuricemia diagnostic criteria, during fetal cardiac development may also increase the risk of CHDs in offspring.

### Maternal Elevated Glucose Levels and CHD Risk

We observed that maternal elevated FPG (>4.35 mmol/L), 1-h PG (>6.14 mmol/L), and 2-h PG (>7.01 mmol/L) levels during early pregnancy were associated with an 18-54% increased risk of CHDs. The thresholds of glucose levels to predict CHDs in our study were lower than the clinical definition of gestational diabetes mellitus (DM) recommended by the World Health Organization as one or more of the following criteria are met: FPG 5.1–6.9 mmol/L, 1-h PG ≥ 10.0 mmol/L or 2-h PG 8.5–11.0 mmol/L (26, 27).

It is well-known that women with diabetes are at a significantly increased risk of having offspring with CHDs. Maternal hyperglycemia is the most common medical condition experienced by pregnant women and is associated with a 2-5-fold increase in CHDs independent of genetic contributors (28–30). A nationwide cohort study conducted in Denmark found that the association between maternal pregestational DM and increased CHD risk neither changed over time nor differed by diabetes subtype (31). All CHD phenotypes were associated with maternal pregestational DM (RRs ranged from 2.74 to 13.8). The association between CHDs and acute pregestational diabetes complications was robust, suggesting a role for glucose in the causal pathway of CHDs. Clinical data suggest that glucose metabolism during preconception, first trimester, and second trimester is strongly correlated (32). Thus, our results regarding the associations between CHDs and glucose imbalance during early pregnancy are comparable with previous studies. In addition, recent research has shown that pregnant women with elevated blood glucose levels, even those below the threshold of diagnosable diabetes, were more likely to have babies with CHDs (33). This retrospective study included 19,171 mother-child dyads and found that plasma glucose levels during early pregnancy were associated with an increased risk of CHDs in expectant mothers without diabetes (aOR = 1.08, 95%CI: 1.02-1.13 per 10 mg/dl increase in random first trimester plasma glucose values). The consistency of our results with this study indicates that glucose levels during early pregnancy may be a modifiable risk factor for CHDs in non-DM mothers.

The pathways through which maternal elevated glucose levels induce CHDs are unclear. Previous animal studies suggested that potential mechanisms may include increased oxidative stress levels in the fetal heart (34, 35), apoptosis of heart tissue (36, 37), abnormal regulation of critical pathways in cardiac development (38, 39), and biological behavior variation of cardiac neural crest cell during development under the context of higher glucose levels (40). If the results from animal studies can be generalized to humans, transient elevations in glucose values during critical periods of cardiac development might disturb the cardiac neural crest migration even in embryos of mothers without diabetes and thereby contribute to the risk of CHD in the fetuses (33).

### Effect of Maternal Hypercoagulation on the Risk of CHDs

Decreased INR and APTT and increased PT-A were associated with increased risk of CHDs in the current study. Although the thresholds of INR, APTT, and PT-A were within the current clinical reference intervals, maternal higher coagulation was associated with a 1.17-1.42 times higher risk of having a baby with a CHD.

Although hypercoagulation is a well-known significant risk factor for adult CVD, the potential association between hypercoagulation and CHDs has seldom been noticed in previous studies (41, 42). A recent study found that self-reported maternal clotting disorders (aOR=8.55, 95%CI: 1.51-48.44) and prescriptions for the anticoagulant enoxaparin (aOR = 3.22, 95%CI: 1.01-10.22) were significantly associated with elevated CHD risk (43). Our study suggests that hypercoagulation, instead of anticoagulant use, might be the real risk factor of CHDs. Pregnancy is a hypercoagulable state, or at least a factor in hypercoagulability, due to the physiologically adaptive mechanism in the coagulation and fibrinolytic systems to prevent postpartum bleeding (44). However, when combined with an additional hypercoagulation, the risk of thrombosis or embolism may become substantial due to potential interaction between pregnancy and an acquired or heritable thrombophilia, which may cause adverse experiences (44). Maternal thrombophilia was associated with pre-eclampsia, intrauterine growth restriction, and abruption in previous studies (45). Thus, maternal hypercoagulation may increase CHD risk *via* thrombosis.

### Application of ML in Clinical Data

Although predictive models using ML algorithms have been developed and used for a great variety of diseases, including CVD, such predictive models were seldom used to predict CHDs or other birth defects. In the current study, we adopted EBM to predict CHDs based on 1,127 variables, including both laboratory test results during early pregnancy and self-reported information from questionnaires, to obtain an overall AUC, accuracy, sensitivity, and specificity of 76% (69-83%), 0.65, 0.74, and 0.65, respectively. Three previous studies tried to predict CHD occurrence using the ML algorithms mentioned above (9–11). The previous hospital-based, case-control study achieved a sensitivity of 0.78, specificity of 0.90, accuracy of 0.86, Yuden Index of 0.68, and AUC of 0.87 (9). The other retrospective cross-sectional study obtained an AUC of 0.82 (0.78-0.84), 0.81 (0.78-0.84), and 0.80 (0.77-0.83) for WSVM, Logit, and WRF models, respectively (10).

The predictive values of the models used in previous studies are comparable to ours. However, our study has several advantages and impacts over previous work. First, we conducted a birth cohort study, which is better at determining causal inference than previous studies using a case-control (9) or cross-sectional design (10, 11). All predictors in our study were collected prior to diagnosing any CHD and, therefore, recall bias was minimized accordingly. The cohort study design also illustrates the temporal sequence from predictor to CHD more clearly. Second, our study is the first study to include maternal laboratory test results as predictors of CHDs together with other self-reported variables from questionnaires. These laboratory indicators were routinely collected in clinical settings or hospitals to assist in diagnosis and treatment. However, they have never been considered in any previous studies to predict CHDs. The leverage and effective utilization of routinely collected data in predicting CHDs will be highly cost-effective. Our findings have a high potential for direct clinical application. Third, our prediction of CHD risk factors was based on EBM, which performs better than other ML approaches that were used in many previous studies.

### Clinical Implications

Our results will have several important clinical implications. First, routine laboratory test results may be more important predictors of CHDs than self-reported risk factors from questionnaires. Second, among the multiple routine laboratory test results, maternal UA, glucose, and coagulation function indicators were the most important and consistent predictors of CHDs. Third, elevated UA, glucose, and coagulation levels, even those below the current clinical definition of abnormal, were consistently associated with increased CHDs.

To implement our results in clinical practice, (1) we developed an online predictive tool for CHDs (https://xdeng3.shinyapps.io/CHD_model/). On this website, probabilities of CHDs will be calculated after inputting/importing the values of the top predictors of CHDs. For example, pregnant women with a >50% probability of having a baby with a CHD could be identified and referred for echocardiogram screening. (2) Screening factors with high sensitivity (INR and PT-A) could be used first to identify most potential CHDs cases but would also bring a high false positive rate. (3) More reliable factors, such as UA, APTT, fasting, 1-h, and 2-h PG levels, should be used to make comprehensive medical judgments. Finally, (4) pregnant women with elevated UA, glucose, and coagulation levels, especially those clinically diagnosed, are strongly encouraged to undergo fetal echocardiogram screening to detect CHDs earlier. Early diagnosis of fetal CHDs enables early referral, even prior to delivery of critical CHD patients, to centers with substantial expertise in managing CHDs and can improve newborn outcomes accordingly. In addition, emerging *in utero* therapies provide additional intervention options in the case of early CHD diagnosis.

### Strengths and Limitations

Our study contains several strengths. First, this is a birth cohort study. All predictors were collected before CHD diagnosis and made the association assessment more reliable. This time sequence from exposure to CHDs allowed us to infer the causal associations between the predictors and CHDs in offspring. Second, we are the first study to consider maternal laboratory test results as predictors of CHDs together with other self-reported variables from questionnaires. In addition, we focused on the clinical indicators during early pregnancy, which covers the critical window of fetal heart development. According to our results, these clinical indicators during early pregnancy are essential predictors of CHDs and should not be neglected. Third, we adopted an advanced ML algorithm, EBM, to predict CHDs based on thousands of variables. This algorithm enabled us to detect never-before-considered predictors of CHDs.

Several limitations should also be considered when interpreting our results. First, the incidence of CHDs in our study (2.9%) is higher than the expected incidence in the general population (1%). Because our study population was enrolled in one of China's largest cardiac referral centers, selection bias may be a concern. Therefore, the generalizability of our study may be limited to more severe cases. However, our study enabled the accumulation of an adequate sample size, which increased study power for severe and critical CHDs. In addition, a sensitivity study was conducted by excluding the referral participants, and the results were similar to our original findings. Furthermore, the generalizability of our findings could be verified by a large prospective multicenter study to confirm the roles of the significant clinical predictors on CHDs. Second, genetic variables were not available in our study because they were not routinely collected unless specially prescribed by physicians. However, we included family history of CHDs in the model. Meanwhile, only a small number of susceptible pregnant women accept genetic tests, which are expensive and not covered by insurance in China. In addition, only a tiny fraction of CHDs was attributed to known genetic factors (<20%). Third, attrition is always a concern for cohort studies. However, we achieved a follow-up rate of 100% in the current study. Forth, reporting bias in the questionnaire is another potential concern. We reduced the reporting bias through several strategies, which were introduced in our previous work (13). Fifth, we checked and ensured that the diagnostic criteria, laboratory tests standards, and reporting system did not change during the entire study period for quality assurance and quality control concerns. Finally, our results for selected variables (e.g., maternal 2-h plasma glucose level) were unstable due to the limited number of cases who were exposed to extreme levels. Therefore, these results should be validated in other cohort studies with larger sample sizes.

## Conclusions

We identified the top 35 predictors of CHDs out of 1,127 variables during early pregnancy using ML approaches. The AUC, accuracy, sensitivity, and specificity of our model ranged from 0.65 to 0.76. Maternal UA, glucose, and coagulation levels were the most consistent and significant predictors of CHDs. Thus, their thresholds, even those below the current clinical definition of abnormal for these three predictors, could be used to help develop CHD screening and prevention strategies.

## Data Availability Statement

The raw data supporting the conclusions of this article will be made available by the authors, without undue reservation.

## Ethics Statement

The studies involving human participants were reviewed and approved by Guangdong Provincial People's Hospital Human Subjects Committee (No. 2011120H). The patients/participants provided their written informed consent to participate in this study.

## Author Contributions

YQ, XD, SL, and XL: concept and design. YQ, XD, and IR: drafting of the manuscript. YQ and XD: statistical analysis, had full access to all the data in the study, and take responsibility for the integrity of the data and the accuracy of the data analysis. JC and JZ: administrative, technical, or material support. SL and JZ: supervision. All authors acquisition, analysis, or interpretation of data, critical revision of the manuscript for important intellectual content, and read and approved the final manuscript.

## Funding

This study was supported by grants from the Science and Technology Planning Project of Guangdong Province, China (Nos. 2019B020230003, 2017A070701013, 2017B090904034, and 2017030314109), National Key Research and Development Program (No. 2018YFC1002600), Guangdong Peak Project (No. DFJH201802), Guangdong Provincial Key Laboratory of South China Structural Heart Disease (No. 2012A061400008), National Natural Science Foundation of China (No. 81903287), and Natural Science Foundation of Guangdong Province (Nos. 2018A030313785 and 2018A030313329).

## Conflict of Interest

The authors declare that the research was conducted in the absence of any commercial or financial relationships that could be construed as a potential conflict of interest.

## Publisher's Note

All claims expressed in this article are solely those of the authors and do not necessarily represent those of their affiliated organizations, or those of the publisher, the editors and the reviewers. Any product that may be evaluated in this article, or claim that may be made by its manufacturer, is not guaranteed or endorsed by the publisher.

## References

[1] Collaborators G 2017 CHD. Global, regional, and national burden of congenital heart disease, 1990-2017: a systematic analysis for the Global Burden of Disease Study 2017. Lancet Child Adolesc Heal. (2020) 4:185–200. 10.1016/S2352-4642(19)30402-X 31978374 PMC7645774

[2] Pierpont ME Basson CT Benson DWJ Gelb BD Giglia TM Goldmuntz E. Genetic basis for congenital heart defects: current knowledge: a scientific statement from the American Heart Association Congenital Cardiac Defects Committee, Council on Cardiovascular Disease in the Young: endorsed by the American Academy of Pediatrics. Circulation. (2007) 115:3015–38. 10.1161/CIRCULATIONAHA.106.183056 17519398

[3] Pierpont ME Brueckner M Chung WK Garg V Lacro RV McGuire AL. Genetic basis for congenital heart disease: revisited: a scientific statement from the American Heart Association. Circulation. (2018) 138:e653-711. 10.1161/CIR.0000000000000606 30571578 PMC6555769

[4] Jenkins KJ Correa A Feinstein JA Botto L Britt AE Daniels SR. Noninherited risk factors and congenital cardiovascular defects: current knowledge: a scientific statement from the American Heart Association Council on Cardiovascular Disease in the Young: endorsed by the American Academy of Pediatrics. Circulation. (2007) 115:2995–3014. 10.1161/CIRCULATIONAHA.106.183216 17519397

[5] Cowan JR Ware SM. Genetics and genetic testing in congenital heart disease. Clin Perinatol. (2015) 42:373-93. 10.1016/j.clp.2015.02.009 26042910

[6] Shameer K Johnson KW Glicksberg BS Dudley JT Sengupta PP. Machine learning in cardiovascular medicine: are we there yet? Heart. (2018) 104:1156–64. 10.1136/heartjnl-2017-311198 29352006

[7] Hoodbhoy Z Jiwani U Sattar S Salam R Hasan B Das JK. Diagnostic accuracy of machine learning models to identify congenital heart disease: a meta-analysis. Front Artif Intell. (2021) 4:708365. 10.3389/frai.2021.708365 34308341 PMC8297386

[8] Mullen M Zhang A Lui GK Romfh AW Rhee J-W Wu JC. Race and genetics in congenital heart disease: application of iPSCs, omics, and machine learning technologies. Front Cardiovasc Med. (2021) 8:635280. 10.3389/fcvm.2021.635280 33681306 PMC7925393

[9] Li H Luo M Zheng J Luo J Zeng R Feng N. An artificial neural network prediction model of congenital heart disease based on risk factors: a hospital-based case-control study. Medicine. (2017) 96:e6090. 10.1097/MD.0000000000006090 28178169 PMC5313026

[10] Luo Y Li Z Guo H Cao H Song C Guo X. Predicting congenital heart defects: a comparison of three data mining methods. PLoS ONE. (2017) 12:e0177811. 10.1371/journal.pone.0177811 28542318 PMC5443514

[11] Rani S Masood S. Predicting congenital heart disease using machine learning techniques. J Discret Math Sci Cryptogr. (2020) 23:293–303. 10.1080/09720529.2020.1721862

[12] Boyd PA Haeusler M Barisic I Loane M Garne E Dolk H. Paper 1: the EUROCAT network–organization and processes. Birth Defects Res A Clin Mol Teratol. (2011) 91(Suppl 1):S2-15. 10.1002/bdra.20780 21384531

[13] Qu Y Lin S Zhuang J Bloom MS Smith M Nie Z. First trimester maternal folic acid supplementation reduced risks of severe and most congenital heart diseases in offspring: a large case control study. J Am Hear Assoc. (2020) 9:e015652. 10.1161/JAHA.119.015652 32613868 PMC7670504

[14] Chawla NV Bowyer KW Hall LO Kegelmeyer WP. SMOTE: synthetic minority over-sampling technique. JAIR. (2002) 16:321–57. 10.1613/jair.95324088532

[15] Nori H Jenkins S Koch P Caruana R. InterpretML: A Unified Framework for Machine Learning Interpretability (2019).

[16] Ren Z Zhu J Gao Y Yin Q Hu M Dai L. Maternal exposure to ambient PM10 during pregnancy increases the risk of congenital heart defects: evidence from machine learning models. Sci Total Environ. (2018) 630:1–10. 10.1016/j.scitotenv.2018.02.181 29471186

[17] Díaz-Francés E Rubio FJ. On the existence of a normal approximation to the distribution of the ratio of two independent normal random variables. Stat Pap. (2013) 54:309–23. 10.1007/s00362-012-0429-2

[18] Ke B Zeng Y Zhao Z Han F Liu T Wang J. Uric acid: a potent molecular contributor to pluripotent stem cell cardiac differentiation *via* mesoderm specification. Cell Death Differ. (2019) 26:826–42. 10.1038/s41418-018-0157-9 30038385 PMC6461775

[19] Rodríguez-Hernández JL Rodríguez-González F Riaño-Ruiz M Martínez-Quintana E. Risk factors for hyperuricemia in congenital heart disease patients and its relation to cardiovascular death. Congenit Heart Dis. (2018) 13:655–62. 10.1111/chd.12620 30066365

[20] Dearth JC Tompkins RB Giuliani ER Feldt RH. Hyperuricemia in congenital heart disease. Am J Dis Child. (1978) 132:900–2. 10.1001/archpedi.1978.02120340076016 685909

[21] Muiesan ML Agabiti-Rosei C Paini A Salvetti M. Uric acid and cardiovascular disease: an update. Eur Cardiol. (2016) 11:54–9. 10.15420/ecr.2016:4:2 30310447 PMC6159425

[22] Chang C-C Wu C-H Liu L-K Chou R-H Kuo C-S Huang P-H. Association between serum uric acid and cardiovascular risk in nonhypertensive and nondiabetic individuals: the Taiwan I-Lan Longitudinal Aging Study. Sci Rep. (2018) 8:5234. 10.1038/s41598-018-22997-0 29588485 PMC5869680

[23] Hao Y Li H Cao YP Chen YW Lei MY Zhang TY. Uricase and horseradish peroxidase hybrid CaHPO4 nanoflower integrated with transcutaneous patches for treatment of hyperuricemia. J Biomed Nanotechnol. (2019) 15:951-65. 10.1166/jbn.2019.2752 30890227

[24] Virdis A Masi S Casiglia E Tikhonoff V Cicero AFG Ungar A. Identification of the uric acid thresholds predicting an increased total and cardiovascular mortality over 20 years. Hypertension. (2020) 75:302-8. 10.1161/HYPERTENSIONAHA.119.13643 31813345

[25] Chang FM Chow SN Huang HC Hsieh FJ Chen HY Lee TY. The placental transfer and concentration difference in maternal and neonatal serum uric acid at parturition: comparison of normal pregnancies and gestosis. Biol Res Pregnancy Perinatol. (1987) 8:35-9. 3580446

[26] Metzger BE Gabbe SG Persson B Buchanan TA Catalano PA Damm P. International association of diabetes and pregnancy study groups recommendations on the diagnosis and classification of hyperglycemia in pregnancy. Diabetes Care. (2010) 33:676–82. 10.2337/dc10-0719 20190296 PMC2827530

[27] Diagnostic criteria and classification of hyperglycaemia first detected in pregnancy: a World Health Organization Guideline. Diabetes Res Clin Pract. (2014) 103:341–63. 10.1016/j.diabres.2013.10.012 24847517

[28] Nakano H Minami I Braas D Pappoe H Wu X Sagadevan A. Glucose inhibits cardiac muscle maturation through nucleotide biosynthesis. Elife. (2017) 6:e29330. 10.7554/eLife.29330 29231167 PMC5726851

[29] Centers for Disease Control. Perinatal mortality and congenital malformations in infants born to women with insulin-dependent diabetes mellitus–United States, Canada, and Europe, 1940-1988. MMWR Morb Mortal Wkly Rep. (1990) 39:363–5. 2111001

[30] Simeone RM Devine OJ Marcinkevage JA Gilboa SM Razzaghi H Bardenheier BH. Diabetes and congenital heart defects: a systematic review, meta-analysis, and modeling project. Am J Prev Med. (2015) 48:195–204. 10.1016/j.amepre.2014.09.002 25326416 PMC4455032

[31] Øyen N Diaz LJ Leirgul E Boyd HA Priest J Mathiesen ER. Prepregnancy diabetes and offspring risk of congenital heart disease: a nationwide cohort study. Circulation. (2016) 133:2243-53. 10.1161/CIRCULATIONAHA.115.017465 27166384 PMC4890838

[32] Priest JR Yang W Reaven G Knowles JW Shaw GM. Maternal midpregnancy glucose levels and risk of congenital heart disease in offspring. JAMA Pediatr. (2015) 169:1112–6. 10.1001/jamapediatrics.2015.2831 26457543 PMC4996656

[33] Helle EIT Biegley P Knowles JW Leader JB Pendergrass S Yang W. First trimester plasma glucose values in women without diabetes are associated with risk for congenital heart disease in offspring. J Pediatr. (2018) 195:275-8. 10.1016/j.jpeds.2017.10.046 29254757 PMC5869072

[34] Wang F Fisher SA Zhong J Wu Y Yang P. Superoxide dismutase 1 *in vivo* ameliorates maternal diabetes mellitus-induced apoptosis and heart defects through restoration of impaired wnt signaling. Circ Cardiovasc Genet. (2015) 8:665-76. 10.1161/CIRCGENETICS.115.001138 26232087 PMC4618088

[35] Wang F Reece EA Yang P. Oxidative stress is responsible for maternal diabetes-impaired transforming growth factor beta signaling in the developing mouse heart. Am J Obstet Gynecol. (2015) 212:650.e1–11. 10.1016/j.ajog.2015.01.014 25595579 PMC4417061

[36] Wu Y Reece EA Zhong J Dong D Shen W Bin Harman CR. Type 2 diabetes mellitus induces congenital heart defects in murine embryos by increasing oxidative stress, endoplasmic reticulum stress, and apoptosis. Am J Obstet Gynecol. (2016) 215:366.e1-366.e10. 10.1016/j.ajog.2016.03.036 27038779 PMC5260663

[37] Wang F Wu Y Quon MJ Li X Yang P. ASK1 mediates the teratogenicity of diabetes in the developing heart by inducing ER stress and inhibiting critical factors essential for cardiac development. Am J Physiol Endocrinol Metab. (2015) 309:E487-99. 10.1152/ajpendo.00121.2015 26173459 PMC4556884

[38] Bohuslavova R Skvorova L Sedmera D Semenza GL Pavlinkova G. Increased susceptibility of HIF-1α heterozygous-null mice to cardiovascular malformations associated with maternal diabetes. J Mol Cell Cardiol. (2013) 60:129-41. 10.1016/j.yjmcc.2013.04.015 23619295

[39] Klimova T Chandel NS. Mitochondrial complex III regulates hypoxic activation of HIF. Cell Death Differ. (2008) 15:660-6. 10.1038/sj.cdd.4402307 18219320

[40] Morgan SC Relaix F Sandell LL Loeken MR. Oxidative stress during diabetic pregnancy disrupts cardiac neural crest migration and causes outflow tract defects. Birth Defects Res Part A Clin Mol Teratol. (2008) 82:453-63. 10.1002/bdra.20457 18435457 PMC5452612

[41] Chan MY Andreotti F Becker RC. Hypercoagulable states in cardiovascular disease. Circulation. (2008) 118:2286-97. 10.1161/CIRCULATIONAHA.108.778837 19029477

[42] Senst B Tadi P Goyal A Jan A. Hypercoagulability. In: StatPearls. Treasure Island, FL: StatPearls Publishing (2020).

[43] Dolk H McCullough N Callaghan S Casey F Craig B Given J. Risk factors for congenital heart disease: the Baby Hearts Study, a population-based case-control study. PLoS ONE. (2020) 15:e0227908. 10.1371/journal.pone.0227908 32092068 PMC7039413

[44] Gresele P. Platelets in Hematologic and Cardiovascular Disorders: A Clinical Handbook. Cambridge: Cambridge University (2008). p. 511.

[45] Greer IA. Thrombophilia: implications for pregnancy outcome. Thromb Res. (2003) 109:73–81. 10.1016/S0049-3848(03)00095-1 12706634

