# Supplementary material for: Using Innovative Machine Learning Methods to Screen and Identify Predictors of Congenital Heart Diseases
Source: Front Cardiovasc Med. 2022 Jan 7;8:797002. doi: 10.3389/fcvm.2021.797002 (PMC8777022; doi:10.3389/fcvm.2021.797002)
Supplement: Supplementary file 2 [file Data_Sheet_1.docx]

Supplementary Material


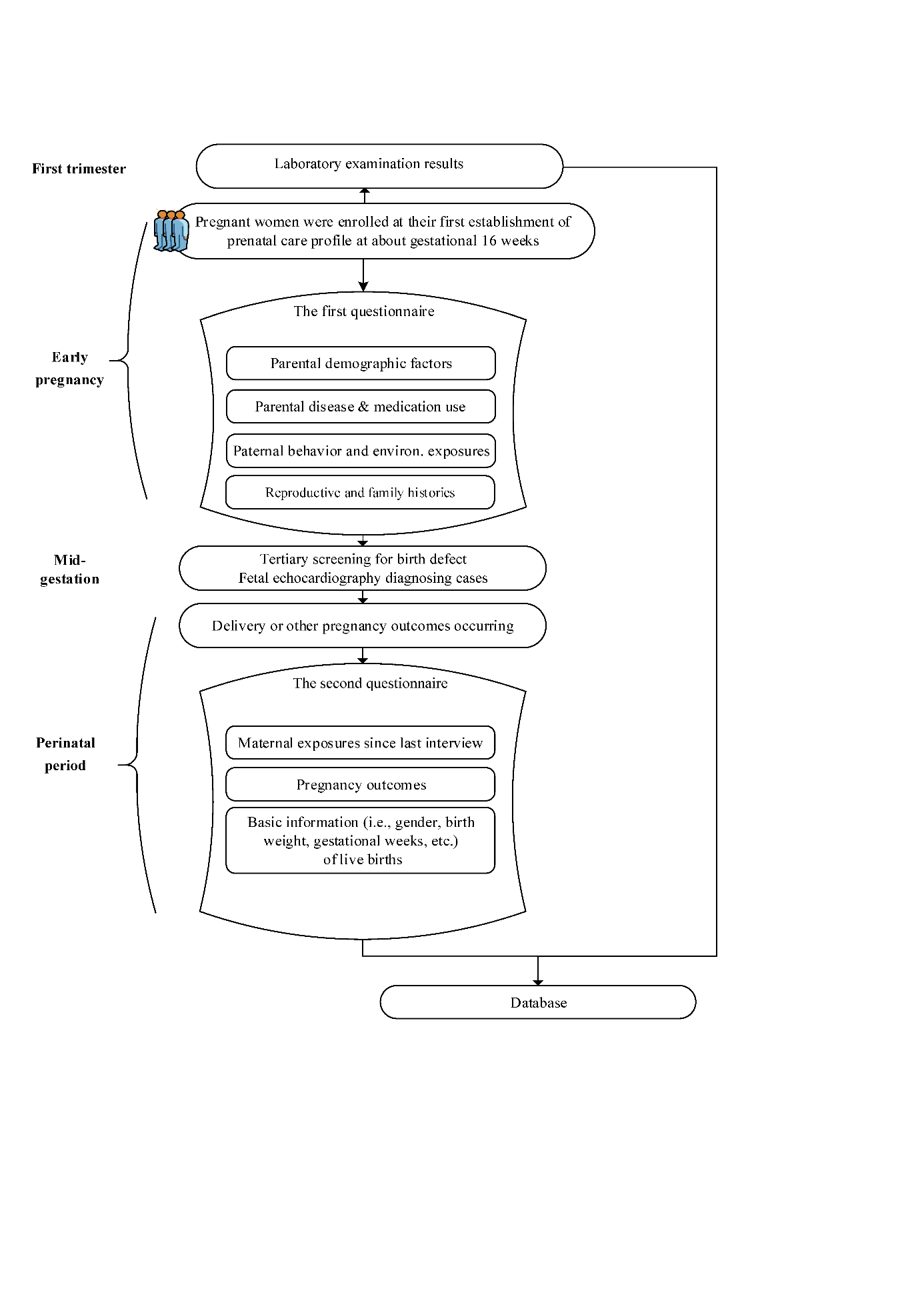


**Supplementary Figure 1.** Flowchart of data collection to predict congenital heart diseases (CHDs) incident, a birth cohort study in Guangzhou, China (N=5,390)

**Supplementary
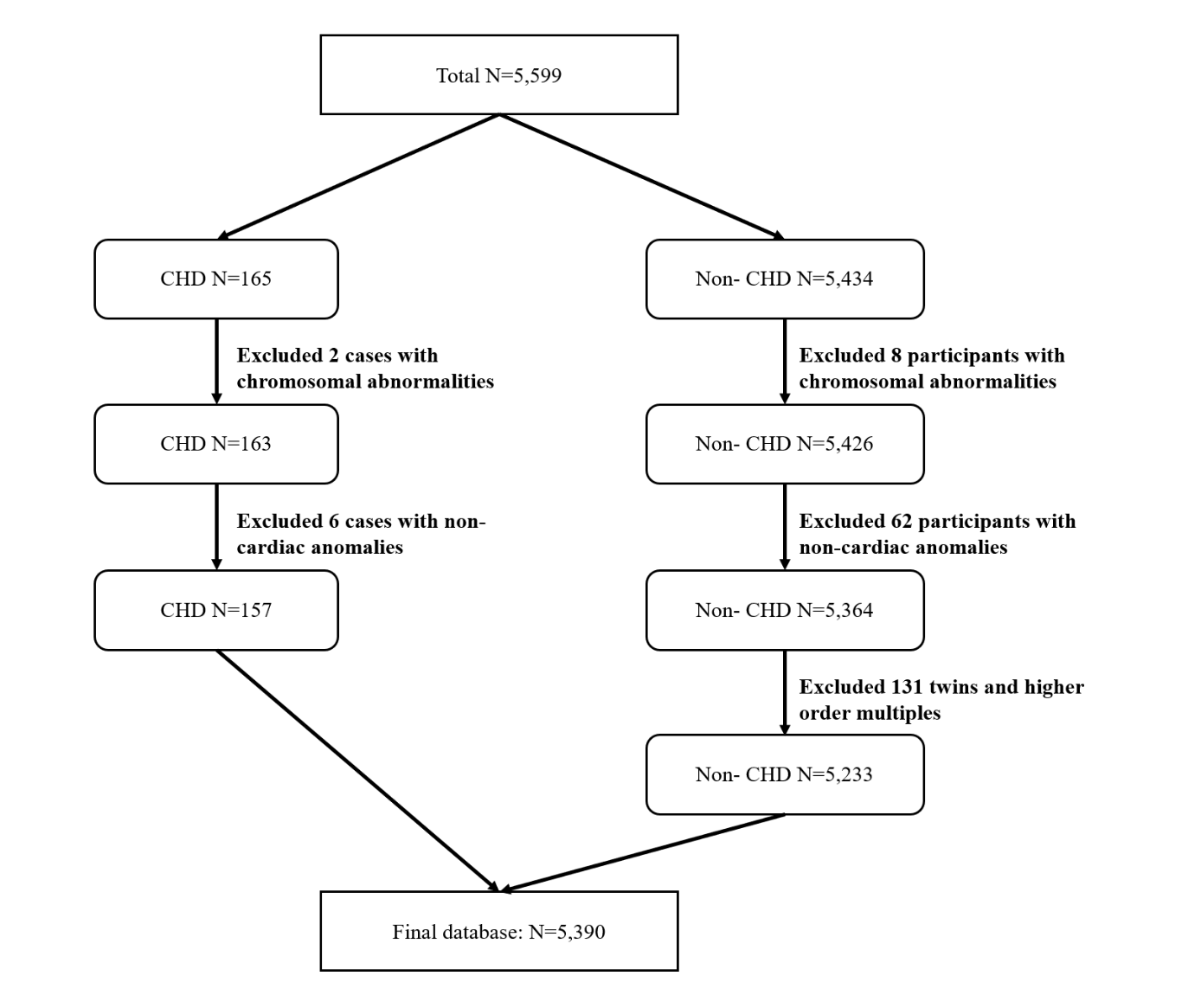
Figure 2.** Flowchart of inclusion and exclusion of participants, a birth cohort study in Guangzhou, China (N=5,390)

**Supplementary Table 1**. Performance comparison of Explainable Boosting Machine, the other six machine learning models and the combined Voting classifier on our data

| **Model** | **Training AUC (95% CI)** | **Testing AUC**  **(95% CI)** |
| --- | --- | --- |
| Explainable Boosting Machine (EBM) | 0.753 (0.709, 0.798) | 0.762 (0.694, 0.830) |
| Random forest | 0.780 (0.738, 0.822) | 0.656 (0.557, 0.754) |
| Gradient Boosting | 0.825 (0.787, 0.864) | 0.697 (0.607, 0.787) |
| Xgboost | 0.835 (0.796, 0.873) | 0.702 (0.616, 0.788) |
| Logistic regression | 0.652 (0.600, 0.704) | 0.680 (0.594, 0.781) |
| ANN | 0.701 (0.653, 0.749) | 0.705 (0.618, 0.792) |
| Naïve Bayesian | 0.662 (0.614, 0.710) | 0.701 (0.605, 0.796) |
| Voting classifier  (Combined the above seven models) | 0.794 (0.752, 0.835) | 0.739 (0.651, 0.828) |

**Supplementary Table 2**. Phenotypes of congenital heart diseases diagnosed in our study

| **Phenotypes of CHDs** | **Number** | **Proportion (%)** |
| --- | --- | --- |
| Ventricular septal defect (VSD) | 37 | 23.6 |
| Transposition of the great arteries (TGA) | 22 | 14.0 |
| Other specified CHDs | 21 | 13.4 |
| Atrial septal defect (ASD) | 21 | 13.4 |
| Pulmonary (valve) stenosis [P(v)S] | 15 | 9.6 |
| Coarctation of aorta (CoA) | 8 | 5.1 |
| Tetralogy of Fallot (TOF) | 7 | 4.5 |
| Total anomalous pulmonary venous connection (TAPVC) | 7 | 4.5 |
| Double-outlet right ventricle (DORV) | 5 | 3.2 |
| Atrioventricular septal defect (AVSD) | 3 | 1.9 |
| Tricuspid anomaly | 3 | 1.9 |
| Ebstein anomaly | 2 | 1.3 |
| Interrupted aortic arch (IAA) | 2 | 1.3 |
| Pulmonary atresia (PA) | 1 | 0.6 |
| Dextrocardia | 1 | 0.6 |
| Aortic-pulmonary window | 1 | 0.6 |
| Aortic arch dysplasia | 1 | 0.6 |
| **Total** | **157** | **100.0** |

**Supplementary Table 3**. Birth outcomes of our birth cohort study participants, by congenital heart diseases, Guangzhou, China (N=5,390)

| **Variables** | **Total** | **CHDs (%)** | **Non-CHDs (%)** | **P-value** |
| --- | --- | --- | --- | --- |
| Total | 5,390 | 157 (2.9) | 5,233 (97.1) |  |
| Pregnancy outcomes |  |  |  |  |
| Live births | 5,354 | 149 (94.9) | 5,205 (99.5) | <0.001 |
| Termination | 13 | 4 (2.5) | 9 (0.2) |  |
| Death after birth | 5 | 4 (2.5) | 1 (0) |  |
| Stillbirth | 17 | 0 (0) | 17 (0.3) |  |
| Spontaneous abortion | 1 | 0 (0) | 1 (0) |  |
| Preterm |  |  |  |  |
| Yes | 382 | 22 (14.0) | 360 (6.9) | 0.001 |
| No | 5,008 | 135 (86.0) | 4,873 (93.1) |  |
| Low birthweight |  |  |  |  |
| Yes | 302 | 26 (16.6) | 276 (5.3) | <0.001 |
| No | 5,088 | 131 (83.4) | 4,957 (94.7) |  |
| IVF-ET |  |  |  |  |
| Yes | 143 | 11 (7.1) | 132 (2.5) | 0.001 |
| No | 5,241 | 145 (92.9) | 5,096 (97.5) |  |

Abbreviations: CHD, congenital heart diseases; IVF-ET, in vitro fertilization, and embryo transfer.

**Supplementary Table 4**. Basic characteristic of our birth cohort study participants, by congenital heart diseases, Guangzhou, China (N=5,390)

| **Variables** | **Total** | **CHDs (%)** | **Non-CHDs (%)** | **P-value** |
| --- | --- | --- | --- | --- |
| Total | 5,390 | 157 (2.9) | 5,233 (97.1) |  |
| **Maternal demographic characteristics** |  |  |  |  |
| Maternal age (years) |  |  |  |  |
| ≥ 35 | 1,480 | 42 (2.8) | 1,438 (97.2) | 0.84 |
| <35 | 3,910 | 115 (2.9) | 3,795 (97.1) |  |
| Maternal education |  |  |  |  |
| ≤12 years | 416 | 26 (6.3) | 390 (93.8) | <0.001 |
| >12 years | 4,969 | 130 (2.6) | 4,839 (97.4) |  |
| Household income (Chines Yuan/person/moth) |  |  |  |  |
| < 3000 | 212 | 10 (4.7) | 202 (95.3) | 0.09 |
| ≥ 3000 | 4,989 | 138 (2.8) | 4,851 (97.2) |  |
| Migrants |  |  |  |  |
| Yes | 634 | 23 (3.6) | 611 (96.4) | 0.25 |
| No | 4,756 | 134 (2.8) | 4,622 (97.2) |  |
| **Maternal main laboratory tests in early pregnancy** |  |  |  |  |
| Gestational weeks at first test | 16.36±2.76 | 16.51±4.40 | 16.35±2.73 | 0.68 |
| Maternal serum uric acid (μmol/L) | 244.02±57.26 | 274.62±71.51 | 243.11±56.54 | <0.001 |
| Maternal glucose levels (mmol/L) |  |  |  |  |
| Fasting glucose | 4.52±0.46 | 4.61±0.39 | 4.52±0.46 | 0.09 |
| 1-hour blood glucose | 7.87±1.69 | 8.07±1.52 | 7.86±1.69 | 0.25 |
| 2-hour blood glucose | 6.81±1.49 | 6.89±1.31 | 6.81±1.49 | 0.65 |
| Maternal coagulation function indicators |  |  |  |  |
| Activated partial thromboplastin time (APTT, seconds) | 33.51±2.82 | 32.91±2.47 | 33.52±2.83 | 0.01 |
| International normalized ratio (INR) | 0.95±0.10 | 0.95±0.05 | 0.95±0.10 | 0.31 |
| Thrombin time (TT, seconds) | 15.03±0.72 | 14.97±0.63 | 15.03±0.72 | 0.29 |
| Prothrombin activity (PT-A) | 109.68±11.25 | 110.55±10.23 | 109.66±11.29 | 0.36 |
| **Maternal medical conditions and obstetric complications** |  |  |  |  |
| Pregestational diabetes and gestational diabetes |  |  |  |  |
| Yes | 987 | 34 (3.4) | 953 (96.6) | 0.27 |
| No | 4,403 | 123 (2.8) | 4,280 (97.2) |  |
| Cardiac disease (including CHDs) |  |  |  |  |
| Yes | 374 | 13 (3.5) | 361(96.5) | 0.50 |
| No | 5,016 | 144 (2.9) | 4,872 (97.1) |  |
| Hypertension |  |  |  |  |
| Yes | 327 | 11 (3.4) | 316 (96.6) | 0.62 |
| No | 5,063 | 146 (2.9) | 4,917 (97.1) |  |
| Preeclampsia |  |  |  |  |
| Yes | 79 | 4 (5.1) | 75 (94.9) | 0.25 |
| No | 5,311 | 153 (2.9) | 5,158 (97.1) |  |
| Renal disease |  |  |  |  |
| Yes | 65 | 3 (4.6) | 62 (95.4) | 0.41 |
| No | 5,325 | 154 (2.9) | 5,171 (97.1) |  |
| Subcutaneous hemorrhage |  |  |  |  |
| Yes | 23 | 1 (4.3) | 22 (95.7) | 0.68 |
| No | 5,367 | 156 (2.9) | 5,211 (97.1) |  |
| Fever in early pregnancy |  |  |  |  |
| Yes | 368 | 12 (3.3) | 356 (96.7) | 0.68 |
| No | 5,022 | 145 (2.9) | 4,877 (97.1) |  |
| Infection in early pregnancy |  |  |  |  |
| Yes | 1222 | 43 (3.5) | 1179 (96.5) | 0.15 |
| No | 4,168 | 114 (2.7) | 4,054 (97.3) |  |
| **Maternal medication use** |  |  |  |  |
| Antibiotics |  |  |  |  |
| Yes | 246 | 5 (2.0) | 241 (98.0) |  |
| No | 5,144 | 152 (3.0) | 4,992 (97.0) |  |
| Tocolytic drugs |  |  |  |  |
| Yes | 1189 | 42 (3.5) | 1147 (96.5) | 0.15 |
| No | 4,201 | 115 (2.7) | 4,086 (97.3) |  |
| Antipyretic analgesic |  |  |  |  |
| Yes | 504 | 19 (3.8) | 485 (96.2) | 0.23 |
| No | 4,886 | 138 (2.8) | 4,748 (97.2) |  |
| Antineoplastic drug |  |  |  |  |
| Yes | 30 | 2 (6.7) | 28 (93.3) | 0.22 |
| No | 5,360 | 155 (2.9) | 5,205 (97.1) |  |
| Vitamin A congeners |  |  |  |  |
| Yes | 322 | 15 (4.7) | 307 (95.3) | 0.06 |
| No | 5,068 | 142 (2.8) | 4,926 (97.2) |  |
| Contraceptive drug |  |  |  |  |
| Yes | 359 | 9 (2.5) | 350 (97.5) | 0.64 |
| No | 5,031 | 148 (2.9) | 4,883 (97.1) |  |
| Chinese medicine |  |  |  |  |
| Yes | 135 | 4 (3.0) | 131 (97.0) | 0.97 |
| No | 5,255 | 153 (2.9) | 5,102 (97.1) |  |
| **Maternal behavior and environment exposures** |  |  |  |  |
| Smoking |  |  |  |  |
| Yes | 34 | 1 (2.9) | 33 (97.1) | 0.99 |
| No | 5,356 | 156 (2.9) | 5,200 (97.1) |  |
| Environmental tobacco smoke exposure |  |  |  |  |
| Yes | 1453 | 47 (3.2) | 1406 (96.8) | 0.39 |
| No | 3,937 | 110 (2.8) | 3,827 (97.2) |  |
| Alcohol consumption |  |  |  |  |
| Yes | 23 | 1 (4.3) | 22 (95.7) | 0.68 |
| No | 5,367 | 156 (2.9) | 5,211 (97.1) |  |
| Living in newly renovated home |  |  |  |  |
| Yes | 462 | 17 (3.7) | 445 (96.3) | 0.31 |
| No | 4,928 | 140 (2.8) | 4,788 (97.2) |  |
| Residing within 50 m of a high traffic roadway |  |  |  |  |
| Yes | 1,541 | 47 (3.0) | 1,494 (97.0) | 0.67 |
| No | 3,843 | 109 (2.8) | 3,734 (97.2) |  |
| Residing within 1.6 km of a pollution source (i.e., chemical plant, waste plant, et al.) |  |  |  |  |
| Yes | 87 | 6 (6.9) | 81 (93.1) | 0.03 |
| No | 5,298 | 150 (2.8) | 5,148 (97.2) |  |

Abbreviations: CHD, congenital heart diseases.

**Supplementary Table 5.** Categories of variables included in our birth cohort study, Guangzhou, China (N=5,390)

| **Variable categories** | **# of Variables** | **Proportion (%)** |
| --- | --- | --- |
| **Self-reported variables from questionnaire** | **379** | 33.63 |
| Maternal socioeconomic status | 12 | 1.06 |
| Maternal exposures during periconceptional periods |  | 0.00 |
| Medical conditions | 17 | 1.51 |
| Medication use | 18 | 1.60 |
| Behavior and environmental exposures | 79 | 7.01 |
| Paternal exposures during periconceptional periods | 86 | 7.63 |
| Maternal exposures from last interview to delivery | 100 | 8.87 |
| Maternal pregnancy complications | 34 | 3.02 |
| Reproductive history | 19 | 1.69 |
| Family history | 14 | 1.24 |
| **Routine maternal laboratory test results** | **699** | 62.02 |
| Serum test | 270 | 23.96 |
| Whole blood test | 154 | 13.66 |
| Routine urinalysis | 84 | 7.45 |
| Plasma test | 82 | 7.28 |
| Cervical vaginal swab test | 24 | 2.13 |
| Vaginal secretion test | 24 | 2.13 |
| Routine stool test | 20 | 1.77 |
| Sputum test | 12 | 1.06 |
| Extraction liquid inspection | 11 | 0.98 |
| Blood sugar test | 5 | 0.44 |
| Drainage test | 5 | 0.44 |
| Throat swab test | 3 | 0.27 |
| Others | 5 | 0.44 |
| **Basic information and outcomes of infants** | **49** | 4.35 |
| **Total** | **1,127** | **100** |

**Supplementary
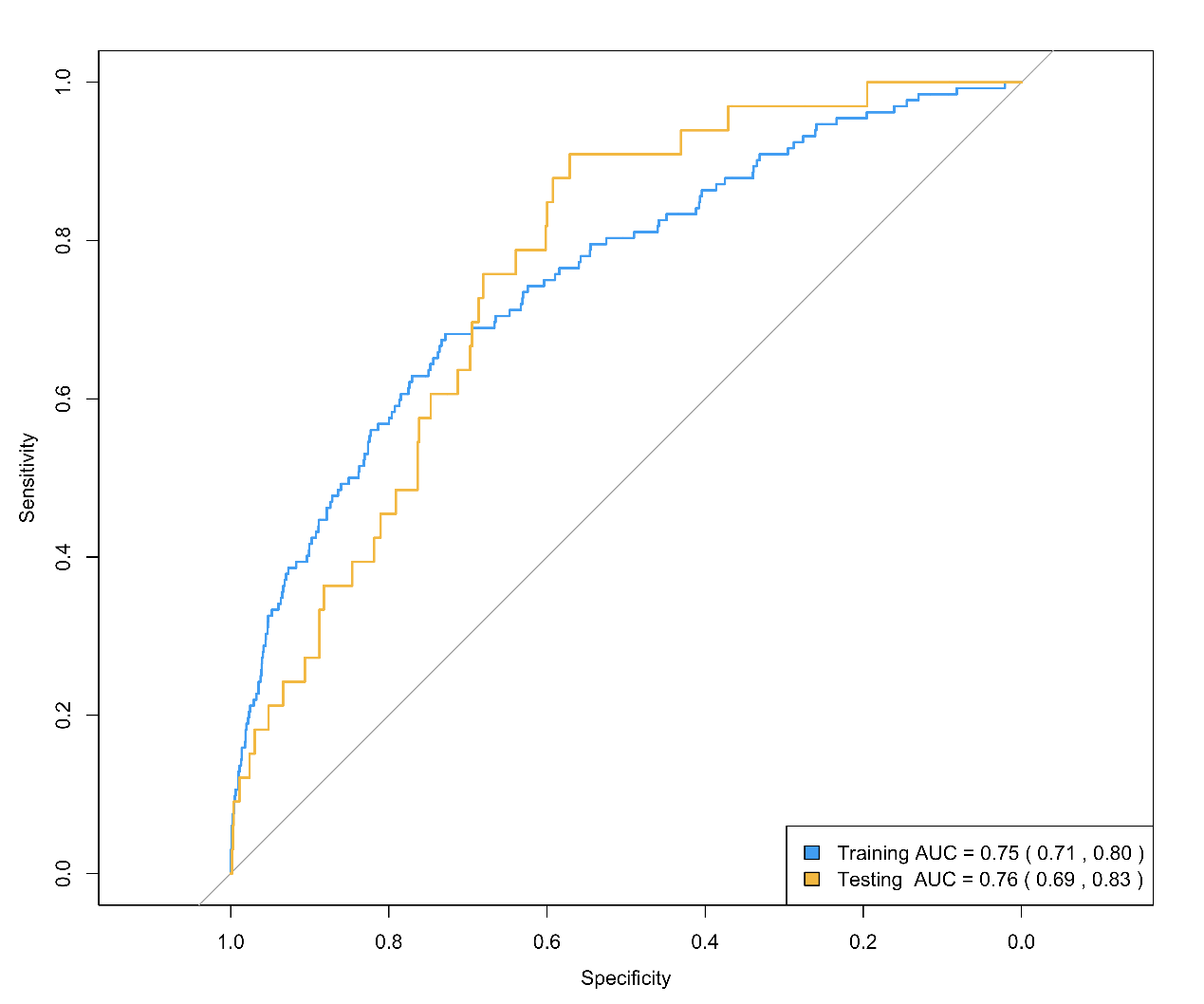
Figure 3**. Results of the ROC analysis for prediction of congenital heart diseases incident, a birth cohort study in Guangzhou, China (N=5,390)

**
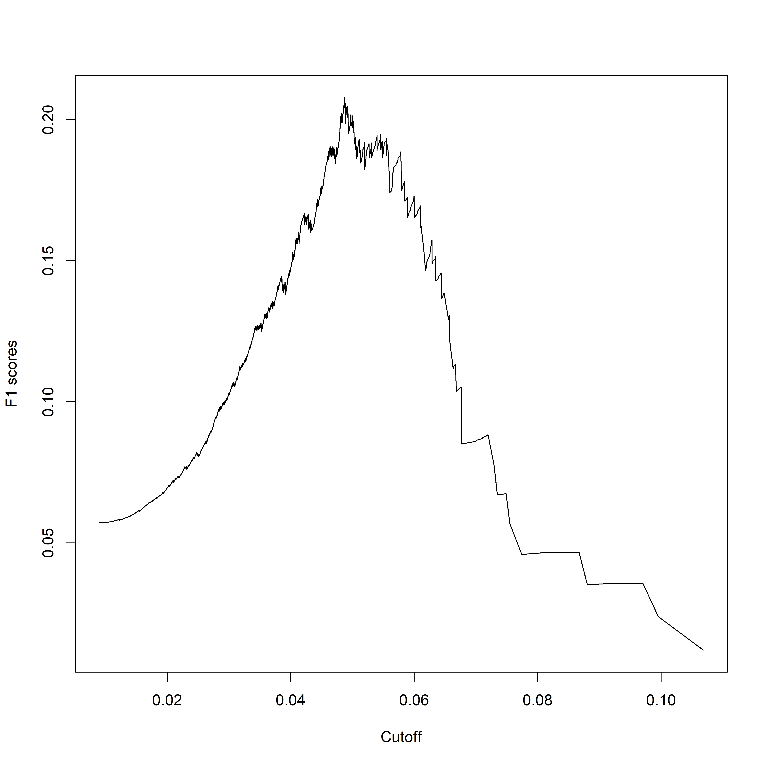
**

**Supplementary Figure 4.** F1 score of our Explainable Boosting Machine


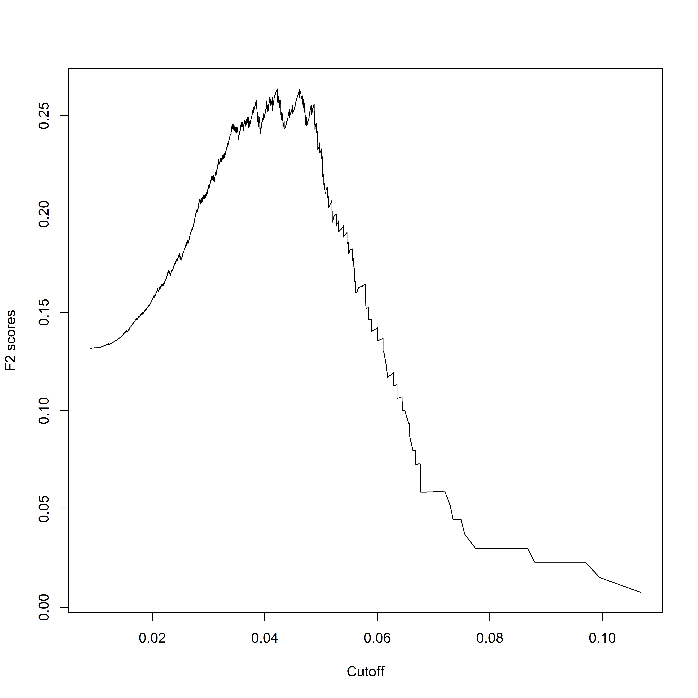


**Supplementary Figure 5.** F2 score of our Explainable Boosting Machine


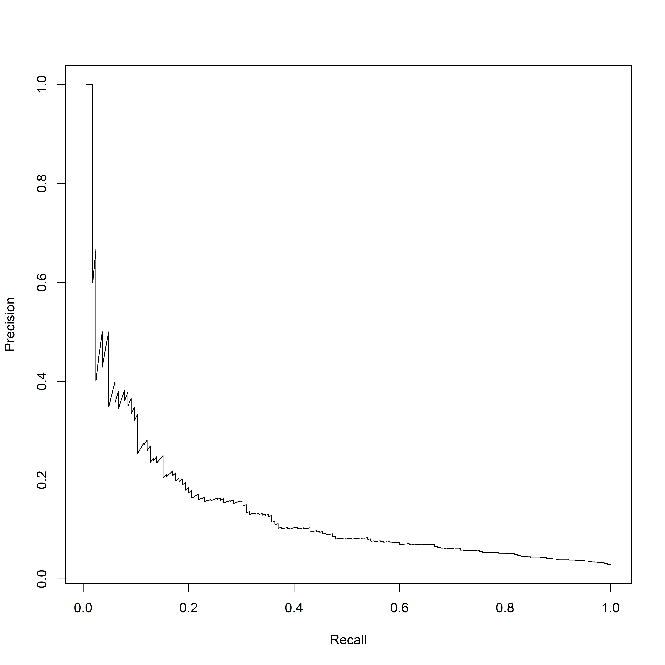


**Supplementary Figure 6.** The Precision-Recall curve of our Explainable Boosting Machine

**Supplementary Table 6.** Scores and definitions of the top 35 predictors for congenital heart diseases in offspring, Guangzhou, China (N=5,390)

| **Original variables** | **Variable names** | **Scores** | **Definitions** |
| --- | --- | --- | --- |
| A6733 | APTT | 0.184894 | Plasma_ Activated Partial Thromboplastin Time |
| A610 | GLUC | 0.181036 | Fasting blood glucose level |
| A5173 | EO% | 0.149456 | Whole blood_ Eosinophil% |
| A4102 | MONO# | 0.119709 | Whole blood_ monocyte |
| A295 | 2HGLUC | 0.080561 | 2-hour blood glucose |
| A277 | 1HGLUC | 0.080421 | 1-hour blood glucose |
| A2413 | OTHERS | 0.0771 | Urine_ Other particulate count |
| A9850 | URIC | 0.067427 | Serum_ Uric acid |
| A4579 | LYMPH% | 0.062522 | Whole blood_ Lymphocyte ratio |
| A5503 | PDW | 0.058746 | Whole blood_ Platelet distribution width |
| A1378 | UC | 0.037151 | Urine_ Casts |
| A5071 | BASO# | 0.032663 | Whole blood_ Basophil count |
| A4630 | LYMPH# | 0.028519 | Whole blood_ Lymphocyte count |
| A3586 | HbA2Hb | 0.021323 | Whole blood_ Hb A2 |
| A775 | WBC | 0.021249 | Urine_ While blood cell |
| A6610 | INR | 0.014892 | Plasma_ International normalized ratio (INR) |
| A4834 | MCV | 0.011457 | Whole blood_ Mean red blood cell volume |
| A6829 | TT | 0.009546 | Plasma_ Thrombin time |
| A3415 | Total | 0.006076 | Urine_ Total particulate count |
| A6076 | NEUT% | 0.00588 | Whole blood_ Neutrophil ratio |
| A7894 | GGT | 0.004606 | Serum_ γ-glutamyl transpeptidase |
| A5329 | HbA1c | 0.004412 | Whole blood_ HbA1c |
| A9811 | UREA | 0.00424 | Serum_ Urea |
| ABORTION1 | AH | 0.003844 | Abortion history |
| A5224 | EO# | 0.003257 | Whole blood_ Eosinophil count |
| A2584 | UEC | 0.003031 | Urine_ Epithelial cells |
| A4000 | MONO% | 0.002834 | Whole blood_ Monocyte ratio |
| A6865 | PT | 0.002586 | Plasma_ Prothrombin activity |
| A10552 | TBA | 0.002309 | Serum_ Total bile acid |
| A7207 | PT | 0.002215 | Plasma_ Prothrombin time |
| A8341 | Co2CP | 0.001973 | Co2_CP |
| A4897 | MPV | 0.001909 | Whole blood_ Mean platelet volume |
| A6127 | NEUT# | 0.001781 | Whole blood_ Neutrophil count |
| A2962 | NonLysed | 0.001455 | Urine_ Non-Lysed Red Blood Cell% |
| Binfection2 | ENEUT | 0.000232 | Elevated neutrophil |
